# Blood Pressure and Fibrinogen Responses to Mental Stress as Predictors of Incident Hypertension over an 8-Year Period

**DOI:** 10.1007/s12160-016-9817-5

**Published:** 2016-07-11

**Authors:** Andrew Steptoe, Mika Kivimäki, Gordon Lowe, Ann Rumley, Mark Hamer

**Affiliations:** 1 Department of Epidemiology and Public Health, University College London, 1-19 Torrington Place, London, WC1E 6BT UK; 2 Institute of Cardiovascular and Medical Sciences, University of Glasgow, Glasgow, Scotland; 3 National Centre for Sport & Exercise Medicine, Loughborough University, Loughborough, UK

**Keywords:** Stress reactivity, Stress recovery, Allostatic load, Inflammation

## Abstract

**Background:**

Heightened blood pressure (BP) responses to mental stress predict raised BP levels over subsequent years, but evidence for associations with incident hypertension is limited, and the significance of inflammatory responses is uncertain.

**Purpose:**

We investigated the relationship between BP and plasma fibrinogen responses to stress and incident hypertension over an average 8-year follow-up.

**Method:**

Participants were 636 men and women (mean age 59.1 years) from the Whitehall II epidemiological cohort with no history of cardiovascular disease and hypertension. They performed standardized behavioral tasks (color/word conflict and mirror tracing), and hypertension was defined by clinic measures and medication status.

**Results:**

Of participants in the highest systolic BP reactivity tertile, 29.3 % became hypertensive over the follow-up period compared with 16.5 % of those in the lowest tertile, with an odds ratio of 2.02 (95 % CI 1.17–3.88, *p* = 0.012) after adjustment for age, sex, grade of employment, body mass index, smoking, alcohol consumption, physical activity, follow-up time, subjective stress response, perceived task difficulty, perceived task engagement, and baseline BP. Similar associations were observed for diastolic BP reactivity (odds ratio 2.05, 95 % CI 1.23–3.40, *p* = 0.006) and for impaired systolic BP post-stress recovery (odds ratio 2.06, 95 % CI 1.19–3.57, *p* = 0.010). Fibrinogen reactions to tasks also predicted future hypertension in women (odds ratio 2.64, 95 % CI 1.11–6.30, *p* = 0.029) but not men.

**Conclusions:**

These data suggest that heightened cardiovascular and inflammatory reactivity to mental stress is associated with hypertension risk, and may be a mechanism through which psychosocial factors impact on the development of hypertension.

## Introduction

The role of psychological stress in the etiology of hypertension has been investigated using two main approaches. The first is observational epidemiology, measuring the associations between exposure to psychological stress and the development of hypertension [1]. The second is the mechanistic approach, studying the impact of acute mental stress on cardiovascular function. A number of longitudinal studies have demonstrated that heightened blood pressure (BP) reactivity to acute stress predicts increases in tonic BP levels over time [2]. The theory underlying these investigations is that individual differences in acute stress reactivity measured on a single occasion reflect the person’s habitual response to everyday stress, and that repeated episodes of cardiovascular activation will lead to fixed hypertension.

Research relating BP stress reactivity with incident hypertension, as opposed to raised BP within the normal range, is relatively limited [3–6], and some studies have relied on self-reported hypertension which may be inaccurate [7]. Associations between heightened BP reactivity to stress and incident hypertension have been positive, but modest in size. One reason may be the use of standard arm cuffs to assess BP reactivity; measures can be obtained only once every 1–2 min with this method, so estimation of dynamic responses is limited compared with assessments based on beat-by-beat methods [8]. Studies of BP stress reactivity are also vulnerable to reverse causality. People with borderline or high-normal BP show raised stress reactivity [9], and are at increased risk of developing hypertension, so heightened reactivity may simply reflect pre-existing risk for hypertension.

In the present study, we attempted to address these issues by analyzing associations between systolic and diastolic BP reactions to acute mental stress measured using beat-by-beat methods and incident hypertension over a period of nearly 8 years in a longitudinal cohort of more than 600 initially healthy men and women. We took into account other factors that might predict incident hypertension including age, body mass index (BMI), smoking, physical activity, and alcohol consumption. Impaired BP recovery back to baseline levels following stress is an indicator of chronic allostatic load [10], and may also predict raised BP over time, so was assessed along with stress reactivity [11]. Furthermore, we assessed associations between heightened fibrinogen responses to stress and future hypertension.

BP reactivity is only one element of a broader pattern of disturbed physiological responses to stress, including neuroendocrine, inflammatory, and hemostatic responses. We have previously shown that greater cortisol responses to acute stress predict incident hypertension over a 3-year period [12]. Inflammation is also potentially relevant, since inflammatory proteins such as fibrinogen are involved in the development of coronary disease [13, 14]. As the principal substrate for thrombin, fibrinogen plays a pivotal role in the coagulation cascade. It stimulates red blood cell aggregation and is a major determinant of plasma viscosity and whole blood viscosity, elevation of which leads to increased total peripheral resistance and elevated systemic arterial pressure. Fibrinogen affects a number of mechanisms involved in hypertensive arterial remodeling, including proliferation of smooth muscle cells, vascular fibrosis (deposition of collagen, fibronectin, and other extracellular matrix components in the vessel wall), apoptosis, low grade inflammation, and oxidative stress. Fibrinogen also stimulates inflammation by upregulating the expression of cellular adhesion molecules, favoring leukocyte recruitment to the endothelium [15]. Stress-dependent hypertension in mouse models has been shown to trigger inflammatory responses and to augment vasoconstriction and sodium retention, acting synergistically with angiotensin II [16]. Studies of healthy people and individuals with hypertension have found positive associations between the concentration of fibrinogen in the circulation and BP [17, 18]. Fibrinogen is also sensitive to acute mental stress, with increases recorded from blood samples taken immediately after challenging behavioral tasks with only partial recovery 45 min later [19]. A small study found that fibrinogen responses to acute stress predicted changes in ambulatory BP over a 3-year period, but did not investigate hypertension [20]. We therefore assessed associations between heightened fibrinogen responses to stress and future hypertension.

## Methods

### Study Sample

Data were analyzed from two studies of stress reactivity carried out with participants from the Whitehall II epidemiologic cohort using identical mental stress tasks and cardiovascular assessment protocols. The Psychobiology Study was conducted between 2000 and 2001 and involved 237 volunteers [21], while the Heart Scan study was carried out from 2006 to 2008 with 543 volunteers [22]. Hypertension outcome was assessed in 2012–2013, with a mean follow-up period of 7.91 ± (standard deviation) 3.4 years. In both studies, we recruited volunteers of White European origin with no history or objective signs of coronary heart disease and no previous diagnosis or treatment for hypertension, inflammatory diseases, or allergies. Participants were free of any prescribed or over the counter medications at the time of testing. Selection was stratified by grade of employment (current or most recent) to include higher and lower socioeconomic status participants. Participants provided informed consent and ethical approval was obtained through the National Research Ethics Service.

We obtained outcome data on hypertension outcome from 736 (94.4 %) of the original sample. However, 83 individuals had resting BP ≥140/90 mmHg at the time of stress testing, despite having no history or formal diagnosis of hypertension. They were excluded from the analyses to protect against reverse causality, along with 17 individuals with missing data on one or more covariates. The analytic sample therefore comprised 636 people (307 men and 299 women). Comparison between the analytic sample and those excluded indicated that, as expected, the excluded individuals had higher resting systolic BP (139.0 ± 16.3 vs 119.4 ± 12.4 mmHg, *p* < 0.001) and resting diastolic BP (81.7 ± 10.4 vs 71.5 ± 8.9 mmHg, *p* < 0.001). They were also slightly older on average (61.8 ± 9.4 vs 59.1 ± 6.7 years, *p* < 0.001) and had greater BMI (26.8 ± 4.0 vs 25.5 ± 3.8, *p* < 0.001). Importantly, there were no differences in systolic BP, diastolic BP, or fibrinogen stress reactivity between individuals included and excluded from the analytic sample (*p* > 0.36), so the groups did not differ on key exposure variables.

### Mental Stress Testing

Mental stress was induced by two behavioral tasks. The first was a computerized color-word interference task, involving the successive presentation of target color words (e.g., green, yellow), printed in another color [23]. At the bottom of the computer screen were four names of colors printed in incorrect colors. The task was to press a computer key that corresponded to the position at the bottom of the screen of the name of the color in which the target word was printed. The rate of presentation of stimuli was adjusted to the performance of the participant, to ensure sustained demands. The second task was mirror tracing, and the tracing of a star with a metal stylus which could only be seen in mirror image [24]. Each time the stylus came off the star, a mistake was registered and a loud beep was emitted by the apparatus (Lafayette Instruments Corp, Lafayette, IN, USA). Participants were told that the average person completed five circuits of the star in the time available, and were asked to give accuracy priority over speed on both tasks.

### Procedure

Mental stress testing was performed individually in either the morning or afternoon in a light temperature-controlled laboratory, and participants were instructed to refrain from drinking caffeinated beverages or smoking for at least 2 h before the study and not to have performed vigorous physical activity or consumed alcohol the previous evening. Brachial BP was measured at rest with an OMRON HEM 907 digital monitor, after which BP was monitored continuously from the non-dominant hand using a Finapres (Psychobiology Study) or Finometer (Heart Scan Study), and a venous cannula was inserted for the collection of blood samples. After a 30-min rest period, baseline BP was monitored for 5 min, a baseline blood sample was drawn, and subjective stress ratings were obtained on a seven-point scale from 1 = *no stress* to 7 = *maximum stress*. Saliva samples were also collected for the measurement of salivary free cortisol, but findings are not reported here, since the protocol differed slightly between the two studies [12]. Two 5-min behavioral tasks were then administered in a random order. Blood pressure monitoring continued throughout tasks, and blood and saliva samples were obtained immediately after the second task. Participants rated subjective stress during the tasks and also provided ratings of task difficulty and task engagement using seven-point scales. Recovery levels of BP were measured 40–45 min after tasks. Blood samples were collected in EDTA tubes and centrifuged immediately at 2500 rpm for 10 min at room temperature. Plasma was removed from the tube and aliquoted into 0.5 ml portions and stored at −80 °C until analysis. Clottable fibrinogen was measured using an automated Clauss assay in a MDA-180 coagulometer (Oragon Teknika, Cambridge, UK).

### Other Measures

Height and weight were measured for the assessment of BMI, and information on smoking, physical activity and alcohol consumption was obtained by questionnaire. Alcohol units per week were derived from questions about the frequency of drinking a range of alcohol beverages. Participants reported how many times a week they engaged in vigorous physical activity (defined as activity that “makes you feel out of breath” such as running and participation in strenuous sports), the type of activity, and their total amount of weekly walking in minutes. Respondents were specifically asked to report how many minutes they walked outside their home or workplace on weekdays and weekends, which were combined to calculate the total weekly amount. These measures have been related to risk of the metabolic syndrome and inflammation in the full Whitehall II study [25, 26], and also predict all-cause mortality [27]. Participants were divided into sedentary (no vigorous activity) and active groups for analysis.

### Hypertension Assessment

Incident hypertension was measured at a clinic assessment carried out as part of the routine follow-up of the Whitehall II cohort in 2012–2013 [28]. Blood pressure was measured twice in the sitting position after 5 min of rest using an OMRON HEM 907 digital sphygmomanometer, and the average was taken. Participants were also questioned in detail about medication status. We defined hypertension according to the 7th report of the Joint National Committee on Prevention, Detection, Evaluation, and Treatment of High Blood Pressure as physician diagnosis, systolic/diastolic BP ≥140/90 mmHg, or use of antihypertensive medication [29].

### Statistical Analysis

We averaged beat by beat BP readings for the 5-min baseline, the two 5-min tasks, and the 40–45-min post-task recovery period. Systolic and diastolic BP stress reactivity was defined as the difference between mean levels during tasks and baseline. Fibrinogen stress reactivity was defined as the difference between concentrations at baseline and after the task period. Incident hypertension was identified in 143 (22.8 %) participants. We compared the characteristics at the time of stress testing of individuals with and without future hypertension using analysis of variance for continuous variables and Chi^2^ analysis for categorical variables. In multivariable analyses, we included as covariates factors known to be related to stress responsivity including age, gender, socioeconomic status defined by grade of employment, smoking status, BMI, alcohol consumption, physical activity, follow-up time, and the baseline level of the exposure variable (BP or fibrinogen) [21, 30–32]. Time of testing (morning or afternoon) was included as an additional covariate in preliminary analyses but did not relate to outcomes so was not entered in the final models.

In order to compare associations between stress responsivity and hypertension across variables, we divided reactions to tasks and recovery levels into tertiles, but the same pattern of results emerged when continuous units of measurement were used to quantify reactivity and recovery. Associations between stress reactivity and incident hypertension were analyzed using logistic regression, computing the odds of hypertension for each tertile increase in systolic BP reactivity, diastolic BP reactivity, or fibrinogen reactivity, compared with the lowest tertile, after adjustment for covariates. Results are presented as odds ratios with 95 % confidence intervals (CI). We used a similar method to evaluate the prognostic significance of impaired post-stress recovery in BP. Since fibrinogen baseline levels differed in men and women, we analyzed fibrinogen responses separately by sex.

## Results

The study sample had a mean age of 59.1 years at baseline, with moderate BMIs and few smokers (Table 1). Baseline BP was low in this healthy sample, averaging 119.4/71.5 mmHg. The incidence of hypertension was 22.8 % overall and did not differ in participants in the Psychobiology and Heart Scan studies (23.2 and 22.1 %, respectively). The 143 participants who developed hypertension over the follow-up period had higher baseline BP measured by brachial cuff or from the finger (*p* < 0.001) and baseline BMI (*p* = 0.006), though values were still in the healthy range on average. There were no significant differences between future hypertensives and the remainder in age at baseline, grade of employment, smoking, alcohol consumption, physical activity, or follow-up interval, but incident hypertension was more common among men (*p* = 0.006).

**Table 1:** Characteristics of study participants

	Complete sample (*n* = 626)	Normotensive on follow-up (*n* = 483)	Hypertensive on follow-up (*n* = 143)	*p* difference
Men	307 (52.2 %)	238 (49.3 %)	89 (62.2 %)	0.008
Women	299 (47.8 %)	245 (50.7 %)	54 (37.8 %)	
Age (years)	59.1 ± 6.7	58.9 ± 6.6	60.1 ± 7.0	0.055
Grade of employment				
Higher	248 (39.6 %)	190 (39.3 %)	58 (40.6 %)	0.93
Intermediate	232 (37.1 %)	181 (37.5 %)	51 (35.7 %)	
Lower	146 (23.3 %)	112 (23.2 %)	34 (23.8 %)	
Body mass index	25.5 ± 3.9	25.3 ± 3.9	26.3 ± 3.4	0.006
Current smoker	37 (5.9 %)	29 (6.0 %)	8 (5.6 %)	1.00
Alcohol units per week	9.08 ± 8.8	8.90 ± 8.4	9.70 ± 10.0	0.34
Vigorous activity	285 (45.5 %)	225 (46.6 %)	60 (42.0 %)	0.34
Follow-up period (years)	7.91 ± 3.4	7.89 ± 3.4	7.99 ± 3.4	0.77
Systolic BP (mmHg)	119.4 ± 12.4	117.2 ± 12.0	126.7 ± 10.6	<0.001
Diastolic BP (mmHg)	71.5 ± 8.9	70.1 ± 8.7	76.3 ± 8.3	<0.001
Fibrinogen (g/l)				
Men (*n* = 319)	2.94 ± 0.58	2.93 ± 0.60	2.97 ± 0.53	0.62
Women (*n* = 285)	3.15 ± 0.62	3.15 ± 0.63	3.13 ± 0.55	0.85
Stress rating (1–7)	1.41 ± 0.78	1.42 ± 0.79	1.36 ± 0.74	0.41
Systolic BP tasks	147.4 ± 20.7	143.7 ± 19.5	159.7 ± 19.9	<0.001
Systolic BP recovery	127.3 ± 15.5	124.9 ± 15.1	135.4 ± 12.4	<0.001
Diastolic BP tasks	85.6 ± 11.5	83.7 ± 11.0	91.8 ± 10.8	<0.001
Diastolic BP recovery	76.5 ± 10.5	75.1 ± 10.4	81.3 ± 9.7	<0.001
Fibrinogen reactivity				
Men	0.12 ± 0.18	0.12 ± 0.18	0.12 ± 0.19	0.86
Women	0.13 ± 0.22	0.12 ± 0.23	0.19 ± 023	0.061
Stress rating response	2.75 ± 1.5	2.70 ± 1.5	2.92 ± 1.4	0.12
Task difficulty (1–7)	5.66 ± 1.0	5.67 ± 1.0	5.65 ± 1.0	0.84
Task engagement (1–7)	5.66 ± 1.3	5.66 ± 1.3	5.65 ± 1.2	0.92

Mean ± standard deviation or *N* (percent)

Subjective ratings of stress increased markedly in response to tasks, rising from a mean 1.41 to 4.16 on the seven-point scale, and participants reported that the tasks were difficult and involving. The BP responses to tasks were substantial, averaging a 28/14 mmHg increase, while plasma fibrinogen concentration increased by 4–5 % (Table 1). In bivariate analyses, systolic and diastolic BP during tasks and in the post-task recovery period were significantly higher in future hypertensives, while fibrinogen stress responses tended to be higher among women who became hypertensive (*p* = 0.061). There were no differences in subjective measures between participants who remained normotensive or became hypertensive over the follow-up period. The intercorrelations between covariates and stress responsivity measures are summarized in Table 2. The mean increase in systolic BP during stress tests averaged 12.2, 27.0, and 44.6 mmHg in the three reactivity tertiles, while the corresponding increases in diastolic BP averaged 6.7, 13.7, and 21.6 mmHg. Table 3 summarizes the multivariate analyses of cardiovascular and inflammatory stress responses as predictors of incident hypertension. The proportion of participants who became hypertensive was 29.3 % in the higher compared with 16.5 % in the lower systolic BP reactivity tertile, with an odds ratio of 2.02 (95 % CI 1.17–3.48, *p* = 0.012) after adjustment for age, sex, grade of employment, smoking status, alcohol consumption, physical activity, follow-up time, subjective stress response, perceived task difficulty and task engagement, and baseline systolic BP. The odds ratio of hypertension for participants in the higher diastolic BP reactivity tertile was 2.05 (95 % CI 1.23–3.40, *p* = 0.006). The other variables independently associated with incident hypertension in the final model were older age (*p* = 0.004), longer follow-up time (*p* < 0.001), and higher baseline BP (*p* < 0.001). We also found that incident hypertension was associated with systolic BP recovery, with an adjusted odds ratio of 2.06 (95 % CI 1.19–3.57, *p* = 0.010) for individuals whose BP remained in the highest compared with lowest tertile during the recovery period.

**Table 2:** Correlations between variables

	SBP base	SBP Δ	SBP recovery	DBP base	DBP Δ	Fib base men	Fib Δ men	Fib base women	Fib Δ women	Age
SBP base	–	0.15*	−0.08*	0.59*	0.08	0.12*	0.13*	0.15*	0.06	0.25*
SBP Δ		–	0.21*	0.14*	0.71*	0.01	0.13	−0.03	0.17*	0.21*
SBP recovery			–	0.03	0.18*	0.06	−0.01	0.09	0.12	0.12*
DBP base				–	0.09*	0.12*	0.12*	0.14*	−0.01	0.09*
DBP Δ					–	0.01	0.12*	0.04	0.08	0.06
Fib base men						–	0.14*			0.26*
Fib Δ men							–			0.15*
Fib base women								–	−0.03	0.18*
Fib Δ women									–	0.24*
Age										–
	Gender	Grade	BMI	Smoke	Alcohol	VPA	F-up time	Stress	Difficulty	Engagement
SBP base	−0.19*	0.09*	0.17*	−0.05	0.06	−0.05	−0.28*	−0.03	−0.02	−0.01
SBP Δ	0.01	−0.04	−0.06	−0.11*	0.01	0.06	−0.22*	0.11*	−0.04	0.08*
SBP recovery	0.11*	0.13*	0.05	0.02	−0.07	−0.07	−0.16*	−0.01	0.01	0.03
DBP base	−0.15*	0.05	0.25*	0.02	0.14*	−0.03	−0.13*	0.02	0.01	−0.03
DBP Δ	0.10*	−0.03	0.01	−0.06	−0.01	0.01	−0.05	0.13*	−0.01	0.09*
Fib base men		0.12*	0.27*	0.15*	0.01	−0.11	−0.26*	−0.01	0.02	0.03
Fib Δ men		0.09	0.04	0.07	−0.02	−0.01	−0.30*	0.06	−0.01	0.01
Fib base women		0.18*	0.27*	0.08	−0.16*	−0.15*	−0.17*	0.04	0.02	−0.11
Fib Δ women		−0.09	−0.07	−0.03	−0.07	−0.10	−0.23*	0.01	0.01	−0.04
Age	0.07	0.08	−0.04	−0.05	−0.06	−0.16*	−0.72*	0.10*	0.02	−0.07
Gender	–	0.05	0.25*	0.02	−0.16*	−0.11*	−0.14*	0.09*	0.11*	−0.03
Grade		–	−0.01	0.08	−0.17*	−0.08*	−0.03	0.03	−0.03	−0.02
BMI			–	−0.02	0.07	−0.07	−0.03	−0.05	−0.04	−0.01
Smoke					0.12*	−0.05	0.05	−0.03	0.01	−0.09*
Alcohol					–	0.09	0.03	0.01	0.06	0.04
VPA						–	0.18*	−0.03	0.03	0.03
F-up time							–	−0.09*	−0.05	0.02
Stress								–	0.33*	0.12*
Difficulty									–	−0.01

Reference groups: gender = men; grade = higher grade; smoke = non-smokers; VPA = non-active

*SBP* systolic BP, *DBP* diastolic BP, *Fib* fibrinogen, *Base* baseline value, *Δ* reactivity, *VPA* vigorous physical activity, *F-up time* follow-up period, *stress, difficulty, engagement* ratings after tasks

*p < 0.05

**Table 3:** Cardiovascular and inflammatory responses to mental stress and incident hypertension

Exposure variable	Category	Incident hypertension (*N*)	Incident hypertension (%)	Adjusted odds ratio^a^	*p* value
Systolic BP task reactivity	Low	34/206	16.5 %	1	
	Medium	47/211	22.3 %	1.35 (0.78–2.32)	0.28
	High	61/208	29.3 %	2.02 (1.17–3.48)	0.012
Systolic BP recovery	Low	40.206	19.4 %	1	
	Medium	52/207	25.1 %	1.74 (1.03–2.94)	0.037
	High	49/207	23.7 %	2.06 (1.19–3.57)	0.010
Diastolic BP task reactivity	Low	34/205	16.6 %	1	
	Medium	43/212	20.3 %	1.00 (0.58–1.71)	0.99
	High	65/208	31.3 %	2.05 (1.23–3.40)	0.006
Diastolic BP recovery	Low	48/207	23.2 %	1	
	Medium	52/208	25.0 %	1.29 (0.78–2.12)	0.32
	High	42/206	20.4 %	1.05 (0.63–1.76)	0.84
Fibrinogen task reactivity (women)	Low	43/196	21.9 %	1	
	Medium	38/205	18.5 %	1.99 (0.83–4.98)	0.12
	High	50/191	26.2 %	2.64 (1.11–6.30)	0.029

^a^Adjusted for age, sex, grade of employment, body mass index, smoking status, alcohol consumption, physical activity, follow-up time, subjective stress response, perceived task difficulty, perceived task engagement, and baseline value of the exposure variable

In women, a heightened fibrinogen stress response independently predicted hypertension, with an odds ratio of 2.64 (95 % CI 1.11–6.30, *p* = 0.045) adjusted for covariates (see Table 3). The incidence of hypertension was 21.9 % among women in the low reactivity tertile compared with 26.2 % in the high tertile. But we did not find any association between fibrinogen responses to stress and future hypertension in men (odds ratio 1.07, 95 % CI 0.55–2.09, *p* = 0.84).

In sensitivity analyses, we repeated the regressions substituting change in BMI between baseline and follow-up for baseline BMI. The results remained the same, indicating that the associations between cardiovascular reactivity and future hypertension were not mediated by changes in adiposity. All significant effects remained the same when BP and fibrinogen responses were modeled as continuous variables rather than by dividing into tertiles.

## Discussion

Our analyses indicate that heightened systolic and diastolic BP reactivity to standardized mental stress predict risk of incident hypertension over a follow-up period of 8 years in middle-aged and older men and women without a previous history of hypertension or cardiovascular disease. The associations were substantial, with a twofold increased risk among people in the higher compared with lower tertile of BP reactivity, and were independent of age, gender, socioeconomic status, BMI, smoking, physical activity, alcohol intake, and baseline BP levels. The effects were also independent of self-rated stress and subjective appraisal of mental stress tasks. In addition, we found that blunted recovery in systolic BP following stress predicted future hypertension. Acute plasma fibrinogen responses to stress were also positively associated with incident hypertension, but only among women.

These results differ from those in a previous study of a younger subset of Whitehall II cohort members with a mean age of 44.1 years at the time of mental stress testing and followed up over 10 years [3]; hypertension on follow-up was associated with systolic and diastolic BP reactivity, but not after age and baseline BP were taken into account. Another study involving younger participants only found associations between stress reactivity and hypertension in subgroups but not in the total sample [5]. More robust associations may have emerged in this study because of the older age of participants (mean 59.1 years) and higher incidence of hypertension. Studies using problem-solving tasks as mental stressors have reported more consistent findings for systolic than diastolic BP reactivity [4, 7]. Diastolic BP reactions to mental stress are arithmetically smaller than systolic BP reactions, and may not be accurately measured with standard brachial cuffs, since these register <5 % of possible BP readings over a 5-min task period.

This was an observational study, so we cannot conclude that heightened reactivity was a cause of future hypertension. However, we took care to reduce the risk of reverse causality by adjusting analyses for baseline BP level and excluding individuals with a history of diagnosed hypertension as well as those who exhibited elevated resting BP at the time of stress testing. This was done in order to protect against the possibility that undetected hypertension preceded the development of heightened stress reactivity, since it has previously been shown that heightened stress reactivity predicts future hypertension in people with borderline hypertension [33]. Nevertheless, raised BP measured in the laboratory before stress testing may also reflect anticipatory responses to the impending mental stress tests rather than stable differences in tonic BP, so might themselves be regarded as stress responses. Blood pressure levels in anticipation of physical exercise testing have been associated with future hypertension [6]. Consequently, it is possible that the impact of BP stress responses on incident hypertension was conservatively estimated in this study, and that stronger effects might have emerged by including participants with elevated BP at the time of stress testing.

We measured subjective ratings of stress and task appraisals because of their potential contribution to differences in stress reactivity. Singer [34] established that task engagement is a major determinant of physiological responses to mental stress. It is possible that individuals who were more absorbed in the tasks would show greater BP reactivity and be more at risk for hypertension. However, ratings of task involvement and difficulty did not differ between people who were hypertensive or normotensive on follow-up, and the associations with BP reactivity remained significant after controlling for these factors.

In addition to associations with BP reactivity, we also found that impaired recovery after stress predicted incident hypertension. Participants whose BP remained higher relative to baseline 40–45 min after stress tasks had a twofold increase in risk compared with those whose BP returned to baseline levels. This finding is biologically plausible because impaired recovery following stress is one of the hallmarks of chronic allostatic load [10] and has been associated with heightened inflammation and hemostatic activation [35], shorter leukocyte telomere length [36], and type 2 diabetes [37]. Impaired recovery reflects disruption of biological regulatory systems that persist after exposure to mental stress, and may indicate persistent activation contributing to hypertension risk.

Plasma fibrinogen was assessed as an inflammatory marker in this study, and increased in response to mental stress. We found that women with greater fibrinogen responses to stress were at raised risk of future hypertension, but the same pattern was not observed in men. As noted in the “Introduction,” fibrinogen is involved in hemostasis, coagulation, smooth muscle cell proliferation in the arterial wall, and other processes that might contribute to risk of hypertension [15, 38]. The reason for the sex difference is not clear. Fibrinogen concentrations tended to be higher among women throughout the study, and although the magnitude of responses was similar in men and women, the variability in responses was greater in women than men. It is therefore possible that the impact of individual variations in fibrinogen response was more pronounced among women because of this wider range. The sex difference in associations with hypertension may be a chance finding and requires corroboration in future studies.

### Study Limitations

This study was carried out with White European middle-aged and older individuals, and did not include any Black participants. Young Black adults appear to be at raised risk of stress-related hypertension [5], so associations with stress reactivity may vary across ethnic groups [39]. We did not assess exposure to chronic life stress over the follow-up period, and this might influence the extent to which cardiovascular stress reactions are elicited repeatedly over time [40]. We defined hypertension as elevated BP in a clinical session, physician diagnosis, and/or the use of antihypertensive medication. Such a definition may lead to some misclassification, but this method has been used extensively in the Whitehall II where it produces valid results [41, 42], and similar criteria are employed in other epidemiological studies such as the Atherosclerosis Risk in Communities and Framingham Heart Studies [43, 44]. We did not obtain measures of dietary sodium and potassium that are potentially relevant. The study did not include a comparison no-stress control group against which to evaluate fibrinogen responses, and stress testing was only carried out once. However, previous studies have shown no significant changes in other inflammatory markers despite repeated measurement across similar time periods [45], and stability of cardiovascular responses over repeated sessions is high [46]. The analysis of post-stress recovery was limited to measures taken 40–45 min after stress, so did not capture shorter term recovery profiles.

## Conclusions

This study has a number of strengths. It was carried out with a well-characterized sample of healthy older adults whose cardiovascular health has been monitored closely over the past 30 years. Because of this, we were confident that known hypertensives were excluded, as were people with any signs of undetected hypertension at baseline. The follow-up rate was high, with 94 % of potential participants being retained in the prospective analysis. In conclusion, these results show that heightened BP reactions to mental stress are associated with a significant risk of future hypertension, and that impaired cardiovascular recovery following stress and inflammatory responses are also predictors of incident hypertension. The study provides a mechanistic substrate for the role that stress-related factors may play in the development of hypertension [1, 47].
